# Molecular Mechanisms Leading from Periodontal Disease to Cancer

**DOI:** 10.3390/ijms23020970

**Published:** 2022-01-16

**Authors:** Bartosz Kamil Sobocki, Charbel A. Basset, Bożena Bruhn-Olszewska, Paweł Olszewski, Olga Szot, Karolina Kaźmierczak-Siedlecka, Mateusz Guziak, Luigi Nibali, Angelo Leone

**Affiliations:** 1 Scientific Circle of Oncology and Radiotherapy, Medical University of Gdansk, Mariana Smoluchowskiego 17 Street, 80-214 Gdansk, Poland; olga.szot@gumed.edu.pl (O.S.); mateusz.guziak@gumed.edu.pl (M.G.); 2 Department of Biomedicine, Neuroscience and Advanced Diagnostics, Institute of Human Anatomy and Histology, University of Palermo, 90127 Palermo, Italy; charbel.basset@unipa.it; 3 Genetics and Pathology and Science for Life Laboratory, Department of Immunology, Uppsala University, BMC Husargatan 3, 75108 Uppsala, Sweden; bozena.bruhn-olszewska@igp.uu.se; 4 Department of Medical Biochemistry and Microbiology, Uppsala University, BMC Husargatan 3, 75108 Uppsala, Sweden; pawel.olszewski.sci@gmail.com; 5 Department of Surgical Oncology, Medical University of Gdansk, Mariana Smoluchowskiego 17 Street, 80-214 Gdansk, Poland; leokadia@gumed.edu.pl; 6 Centre for Host-Microbiome Interactions, Periodontology Unit, Faculty of Dentistry, Oral & Craniofacial Sciences, King’s College London, London SE1 9RT, UK; luigi.nibali@kcl.ac.uk

**Keywords:** periodontal disease, cancer, tumorigenesis, *Fusobacterium nucleatum*, *Porphyromonas gingivalis*, RANK ligand, immune response

## Abstract

Periodontitis is prevalent in half of the adult population and raises critical health concerns as it has been recently associated with an increased risk of cancer. While information about the topic remains somewhat scarce, a deeper understanding of the underlying mechanistic pathways promoting neoplasia in periodontitis patients is of fundamental importance. This manuscript presents the literature as well as a panel of tables and figures on the molecular mechanisms of *Porphyromonas gingivalis* and *Fusobacterium nucleatum*, two main oral pathogens in periodontitis pathology, involved in instigating tumorigenesis. We also present evidence for potential links between the RANKL–RANK signaling axis as well as circulating cytokines/leukocytes and carcinogenesis. Due to the nonconclusive data associating periodontitis and cancer reported in the case and cohort studies, we examine clinical trials relevant to the topic and summarize their outcome.

## 1. Introduction

Periodontal disease (PD) may occur regardless of age. It is prevalent in Europe in 5–20% of adults aged 35–44 years old and in 40% of the elderly aged 65–74 [1]. It is caused by an inflammatory reaction to dental plaques as a result of an accumulated bacterial biofilm. Initially, the inflammation is confined to the gingiva (gingivitis), but if left untreated, it can develop into periodontitis in more susceptible people, leading to the destruction of the periodontal ligament and alveolar bone, destabilization of the tooth attachment, and eventually tooth loss [2]. The susceptibility to periodontal disease is individual—it depends on possible dysbiosis and immune response to the microbial accumulation, genetics, oral hygiene and suffering from chronic disease [3,4].

Gram-negative species of bacteria, dominating over species present when healthy, are responsible for the dysbiosis in periodontal disease [5].

*Treponema denticola*, *Porphyromonas gingivalis* and *Tanneralla forsythia*, also known as red-complex triad, are common in periodontal disease [5,6]. *Fusobacterium* spp., *Prevotella intermedia*/*nigrescens*, *Porphyromonas gingivalis* (*P. gingivalis*) and *Aggregatibacter actinomycetemcomitans* were described as the most prevalent subgingival pathogens found in patients with chronic periodontal disease that developed periodontal abscesses [7,8]. Periodontal disease also results in the development of more diverse population of microbiota and an overall biomass increase, potentially caused by an increase of different nutrients available to bacteria due to ongoing inflammation and weakened immunity, insufficient to control bacterial proliferation [5]. 

Cytokines IL-1, TNF and PGE-2 intensify the immune response [9] and via the RANKL pathway stimulate osteoclasts and promote bone loss [10]. IL-1beta and PGE-2 in saliva significantly correlate with PD severity and therefore may be used as markers of treatment effectiveness [11]. AIDS and diabetes mellitus negatively influence the manifestation of periodontal disease [8] and worsen the course of the disease due to their immunocompromising effect [9]. Cancer patients undergoing and after chemotherapy/radiotherapy should be aware of the potential risk of exacerbation of their periodontal disease and maintain good oral hygiene and have frequent dental checkup. Because chemotherapy causes immunosuppression and xerostomia, and radiotherapy damages oral tissue, they make cancer patients more susceptible to PD and a more severe course of the PD [12].

Quick detection of PD can be difficult due to minor early symptoms such as painless bleeding during brushing that is often overlooked by patients. Over time the changes (rubor, swelling, different texture) pertaining to the gingiva become noticeable. Due to the damage to the ligament and alveolar bone, the roots of the teeth become exposed and teeth themselves are more mobile, and eventually they can even fall out. The damaged gingiva will form deepened periodontal pockets [9]. Gingivitis can be modified by medication, hormones, lifestyle, smoking, stress; e.g., drugs such as phenytoin, nifedipine or cyclosporine cause gingiva to overgrowth and estrogen worsens the inflammation [8,9].

The American Academy of Periodontology classification system of periodontal and peri-implant diseases and conditions from 2018 consists of the following categories: (1) gingival diseases (biofilm-induced or non-biofilm-induced); (2)periodontitis encompassing the following conditions: (a) necrotizing periodontal diseases, (b) periodontitis, c) periodontitis as a manifestation of systemic disease; (3) other conditions affecting the periodontium comprising the following subdivisions: (a) periodontal abscesses and periodontal-endodontic lesions, (b) periodontal manifestations of systemic diseases and developmental and acquired conditions: systemic diseases or conditions affecting periodontal supporting tissues, (c) mucogingival deformities and conditions, (d) traumatic occlusal forces, (e) tooth- and prosthesis-related factors [13].

Inflammation associated with periodontal diseases affects the whole organism and increases the risk of cardiovascular diseases, progression of diabetes, respiratory infections, pregnancy problems and also rheumatoid arthritis (Table 1) [14,15].

Chronic periodontitis exposes organisms to bacterial endotoxins, enzymes, metabolic by-products and constantly stimulates the immune response and secretion of cytokines, chemokines and prostaglandins [16]. Chronic inflammation inhibits apoptosis, elongates the cell cycle, stimulates proliferation, migration and angiogenesis [17]. Oxidative stress damages the mucosa making it more susceptible to other carcinogens such as tobacco, alcohol, HPV and EBV [17]. All of the aforementioned factors may predispose individuals to the development of head-and-neck squamous cell carcinoma. The potential carcinogenic effect of the inflammation and bacteria present in periodontal disease has been analyzed in connection with the development/progression of oral squamous cell carcinoma (OSCC) in numerous studies [18,19,20,21]. Periodontal disease can induce carcinogenesis in patients with no previous history of OSCC risk factors such as overuse of alcohol, tobacco or HPV infection [22].

Bacteria and cytokines from the oral cavity are carried around the body through circulation. Therefore, periodontal disease may stimulate cancer formation and development in locations relatively distant from the oral cavity [15].

The main question is whether there is a correlation between cancer occurrence and PD. Several large and medium-scale epidemiological studies and meta-analyses seem to give affirmative answers to this question. A large epidemiological study including 73,737 participants showed that PD is associated with approximately a 30% higher risk of breast cancer in postmenopausal women who were current or past smokers [23]. The importance of this study is strengthened by the fact that those individuals had no history of breast cancer at the time of PD onset. Periodontal disease may increase the risk of developing lung cancer [24,25], esophageal and gastric adenocarcinoma [26], serrated polyps and adenomas [27], postmenopausal breast cancer [23], colorectal cancer [28], prostate cancer [29] and pancreatic cancer [30]. In the case of OSCC and pancreatic cancer, the periodontal disease also correlates with a higher mortality [30]. Cancer and cancer treatment may influence periodontal disease as well; e.g., tamoxifen, used in breast cancer treatment, can reduce periodontitis [31], whereas androgen deprivation therapy for prostate cancer can induce periodontal disease [32].

## 2. Molecular Mechanisms Linking Periodontal Disease with Cancer

### 2.1. Molecular Mechanisms of P. gingivalis and F. nucleatum Pathogenesis in Cancer

Recent epidemiological studies point towards a positive increase in the risk of cancer incidence and/or mortality in PD [16]. Dysbiosis occurs in chronic periodontitis reflected by the prevalence of oral pathogens [33]. *P. gingivalis* and *Fusobacterium nucleatum* (*F. nucleatum*) are key microbial pathogens in the pathogenesis of periodontitis [34]. They play a pivotal role in mediating and promoting carcinogenesis [18]. Researchers have taken a particular interest in exploring the involvement of *P. gingivalis* in carcinoma due to its ability to evade the immune system while maintaining a persisting chronic inflammation state in the surrounding environment [35]. Similarly, but to a lesser extent, *F. nucleatum* role in carcinogenesis has been a focal point due to its ability to coaggregate with oral biofilm colonizers and modulating other bacteria’s crossing of the host’s epithelial and endothelial barrier [36,37,38].

#### 2.1.1. Role of *P. gingivalis* in Mediating Cellular Transformation

In this section, we take a closer look at the molecular mechanisms by which *P. gingivalis* modulates the cellular machinery to instigate tumor-like properties and cellular transformation. In an attempt to investigate the underlying pathophysiology of *P. gingivalis* chronic infection in OSCC, long-term infections of *P. gingivalis* in human immortalized oral epithelial cells (HIOEC) over 15 (HIOEC-15) and 23 (HIOEC-23) weeks were established [39]. HIOEC-15 and HIOEC-23 developed slender or anomalous cell shape and exhibited an absence of cell contact inhibition. The ultrastructure of the infected cells was marked by aberrant nucleoli and heterochromatin and weakened cellular junctions highlighted by a paucity of desmosomes, all being morphological characteristics of cancerous cells. Interestingly, plakophilin 1 (PKP1), which stabilizes desmosomes, was decreased in *P. gingivalis*-infected cells and its decrease was previously associated with a poorer prognosis and shorter time to the onset of metastasis in OSCC patients [40]. Tumor-like features were acquired by HIOEC-15 and HIOEC-23, as they showed increased proliferation, migration and invasion. Colony-stimulating factor 1 (CSF1), growth arrest specific 6 (GAS6), friend leukemia virus integration 1 (FLI1), CD274 (also known as B7-H1), programmed cell death 1 ligand 2, also known as B7-DC (PDCD1LG2), colon-cancer-associated transcript 1 (CCAT1) and nicotinamide N-methyltransferase (NNMT), all of which are markers of tumorigenesis, were upregulated in *P. gingivalis*-infected cells. Finally, proMMP9 and activated MMP9, known as markers of cellular invasion, were increased in *P. gingivalis*-challenged cells [39]. The authors postulate that cellular transformation is achieved in the case of HIOEC *P. gingivalis*-infected cells ultimately by the binding of transcription factor FLI1 to the promotor of CCAT1 who is associated with tumor cell migration and proliferation. *P. gingivalis* activation of CD274 and PDCD1LG2 may allow the tumor cells to evade antitumor immune responses. CSF1 upregulation modulates the increase of GAS6 that will bind to TAM (Tyro3, Axl and MerTK) receptor tyrosine kinases (RTKs) to activate several neoplastic responses. *P. gingivalis* activation of NNMT may be associated with the acquisition of cancer stem cells (CSC) properties.

GroEL, a member of the heat shock protein (HSP) 60 family, is considered one of the virulent factors released by *P. gingivalis* [41]. The role of GroEL in promoting neovasculogenesis was investigated in vitro and vivo, albeit unorthodoxly, as neither the cells nor the mice were infected by *P. gingivalis* but only treated or injected with GroEL. Nevertheless, we thought it would still be important to report it as previous studies unveiled an important role for *P. gingivalis*-secreted GroEL in periodontal disease [42,43,44]. GroEL treatment increased tumor size and volume and the mortality rate of transgenic immunodeficient BALB/c mice injected with C26 (mice colon cancer) cells [45]. GroEL induced neoangiogenesis in epithelial progenitor cells (EPC) and promoted their migration and progression by upregulating E-selectin via activation of the PI3K i, p38MAPK and JNK/SAPK pathways and to a lesser extent via the NOS-related pathways [45].

*P. gingivalis* was shown to activate both the JAK/Stat and PI3K/Akt pathways to inhibit the apoptotic intrinsic pathway by preventing mitochondrial membrane depolarization and blocking cytochrome c release followed by downregulation of proapoptotic (caspase 3, caspase 9, Bad and Bax) and upregulation of antiapoptotic genes (survivin, Bcl-2, bcl-XL and Bfl-1) in gingival epithelial cells (GEC) [46,47,48,49,50]. *P. gingivalis* is able to manipulate mitochondrial apoptotic pathways by RgpA adhesin domain peptide A44 translocation into host mitochondria, triggering upregulation of antiapoptotic genes paralleled by a simultaneous downregulation of proapoptotic events [51]. *P. gingivalis* is also able to regulate prosurvival pathways in GEC by inducing GSH antioxidant activity and inhibiting eATP-P2X7 receptor/NADPH-oxidase-mediated intracellular and mitochondrial ROS generation by nucleoside-diphosphate-kinase (Ndk)-eATP consumption, thus blocking P2X7 ligation-mediated apoptosis [52,53]. *P. gingivalis*’s ability to combat oxidative stress could be mediated by a transient surge of the mitochondrial uncoupling protein 2 (UCP2) gene postinfection [52].

A proteomic analysis aimed at investigating cell cycle pathways in *P. gingivalis* challenged GEC, showed that *P. gingivalis*’s FimA fimbriae, through binding to integrin receptors on the GEC surface, may activate the transduction of signaling to modulate proliferation by accelerating the progression through S-phase [54]. Specifically, *P. gingivalis* acts by upregulating cyclin A, cdk4 and cdk6’s expression and activation of cdk2, while downregulating the expression of cyclin D and INK4. p53’s levels and activation were decreased as well as its respective kinases Chk2, CK1 delta, CK1 epsilon and Aurora A. PI3K, PDK1, p70S6K and p90RSK on the other hand were increased while PTEN was inactivated by phosphorylation at s370 and their levels were diminished [54]. Other studies reported a postinfection increase in both cyclin D1 and cyclin E, both involved in promoting the transition from the G1 to S phase, paralleled by a decrease in p21 [55,56]. The discrepancy related to those findings may be due to the use of immortalized human gingival epithelial (IHGE) cells or other types of cells, i.e., human periodontal ligament fibroblasts (PDLF) in the latter studies instead of primary GEC. In addition, *P. gingivalis* induces the production of inflammatory cytokines IL-6, IL-8, sICAM-1 and MCP-1 and their increase may be partially dependent on RgpA-Kgp activity, while the secretion of MIP-1α and IL-1α postinfection were found to be independent of RgpA-Kgp proteinase–adhesin complex [55,57]. This creates an inflammatory environment that is favorable for tumor growth. *P. gingivalis*-infected GEC upregulates the expression of miRNA-203 that exerts its silencing effect on the suppressor of cytokine signaling 3 (SOCS3) and SOCS6, but mainly SOCS3, which leads to an increase in Stat3 and culminates in a host cell cytokine response resulting in increased inflammation, an ideal tumorigenic microenvironment [58]. *P. gingivalis* increases TLR2 signaling in CEG through the downregulation of miR-105. Upon TLR2 increase, subsequent IL-6 and TNF-α production leads to the activation of NF-kB, which promotes proinflammation, once again creating an adequate tumor microenvironment [59].

#### 2.1.2. Role of *P. gingivalis* and *F. nucleatum* in Exacerbating Malignancy

In contrast to the previous section, this subdivision focuses on the molecular mechanisms involved in *P. gingivalis* and/or *F. nucleatum*-challenged malignant cells and tissues. *P. gingivalis* through its virulent factor gingipain promotes colorectal cancer (CRC) proliferation and progression by activating the MAPK/ERK pathway and upregulating the transcription of KRAS, BRAF, MEK2, ERK2, c-fos and AP1 [60]. *P. gingivalis* promotes invasion in OSCC by the activation of PAR2/PAR4/NF-kB, p38/HSP27 and ERK1–Ets1 pathways leading to an increase in proMMP9 secretion that is cleaved into its active form by gingipain [61,62,63].

*P. gingivalis* altered fatty acid (FA) metabolism in an oral carcinoma mouse model and promoted tumor growth possibly by de novo FA synthesis pathways reflected by the upregulation of FASN and ACC1 [64]. Coinfection of *P. gingivalis* and *F. nucleatum* was shown to orchestrate an inflammatory response reflected by an increase in TNF-α and IL-1β [65]. The same aforementioned oral carcinoma mouse model was used but was challenged with a coinfection of *P. gingivalis* and *F. nucleatum*, which led to tumor growth, invasion and proliferation [18]. TLR2 and TLR4, but mainly TLR2, induced the increase of IL-6, which most likely activated NF-kB and STAT3; the latter leading to the transcription of cyclin D1, promoting proliferation. Moreover, *F. nucleatum* infection, not *P. gingivalis*, in OSCC cell lines led to an increase in TNF-α, cyclin D1 and heparanase, while both bacteria led to an increase in MMP9 [18].

*P. gingivalis*-infected OSCC cells develop chemoresistance through the activation of Notch1 (notch intracellular domain, NICD) and upregulation of Hes1 and Hey2 genes, resulting in CSC-like properties [66]. *P. gingivalis*-challenged OSCC cells exhibited higher invasiveness and metastatic potential, reflected by an IL-8-mediated increase in MMP-1, MMP-2, MMP-7, MMP-9 and MMP-10 [66,67,68]. IL-8 plays an important role in EMT induction [69]. EMT phenotype in OSCC is marked by a decrease in epithelial marker cytokeratin 13 (Ck13) and an increase in mesenchymal markers N-cadherin and α-SMA and epithelial suppressors transcription factors snail, slug and twist [68]. OSCC cells acquired CSC properties and stemness marked by an increase in CD133 and CD44. Studies report an acquired chemoresistance in *P. gingivalis*-challenged OSCC that could be mediated by an increase in IL-6 and aberrant expression of inflammatory cytokines including IL-2, VEGF and TNF-α [70]. A slower proliferation rate in *P. gingivalis*-infected OSCC could be due to a decrease in cyclin D1 and cdk4 and an increase in cell cycle suppressor p21, which induces cycle arrest at G1 [68,71]. The decrease in proliferation rate correlates directly with an increase in ROS-driven autophagy in OSCC cells, marked by an increase in LC3-II and Atg-5–Atg-12 complex, both involved in mediating autophagy, which may be controlled by *P. gingivalis*’s activation of the PI3K–autophagy axis [71].

*P. gingivalis*, *F. nucleatum* and *T. denticola* were shown to increase migration, invasion and stemness and promote an aggressive phenotype of OSCC via the upregulation of integrin α V [34]. The authors explored the mechanism behind those carcinogenic features specifically for *Treponema denticola* (*T. denticola*), however, as those oral pathogens behave similarly, we thought it would be worth mentioning the pathways discovered for *T. denticola* as they may also be exploited by *P. gingivalis* and *F. nucleatum*. *T. denticola* was shown to promote migration and stemness through the TL4/MyD88-mediated activation of integrin α V/FAK signaling [34].

*F. nucleatum* promotes tumor growth and proliferation in vivo and in vitro in CRC, via FadA-binding to E-cadherin and the activation of the β-catenin pathway [72]. FadA binds to region 3 of the extracellular domain 5 (EC5) of E-cadherin, which in turn gets activated and internalized by clathrin and activates the β-catenin that translocated to the nucleus, and activates inflammatory genes NF-kB1 and NF-kB2, cytokines IL-6, IL-8 and IL18, oncogenes Myc and Cyclin D1, transcription factors LEF/TCF and Wnt genes WNT7a, WNT7b and WNT9a [72]. Note that the clathrin-dependent pathways control the inflammatory response only.

**Figure 1:**
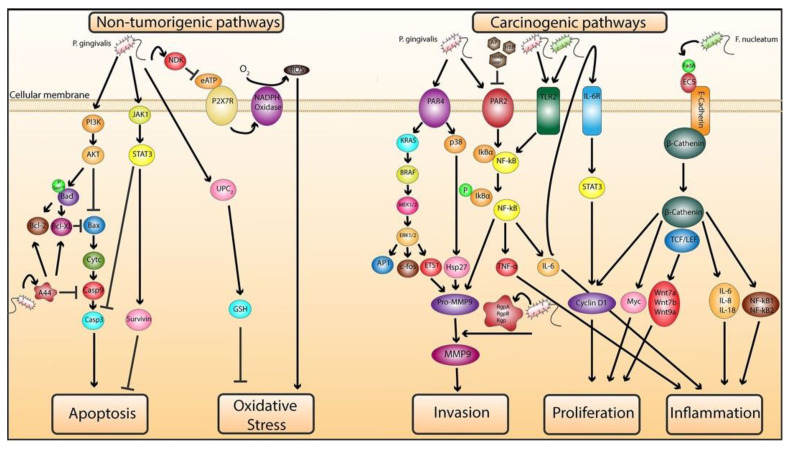
Proposed molecular mechanisms of *P. gingivalis* and *F. nucleatum*-mediated tumorigenesis in tumorous and nontumorous cells. Nontumorigenic pathways: (**left**). *P. gingivalis* promotes antiapoptosis by activating JAK1/STAT3 and PI3K/AKT prosurvival signaling pathways to inhibit caspase3 (casp3) and activate survivin. *P. gingivalis*-mediated activation of PI3K/AKT leads to the phosphorylation of Bad at serine residue 136 and its activation results in its dissociation from antiapoptotic Bcl2 and Bcl-XL proteins, which enhances the antiapoptotic effect of the Bcl2 family reflected by the inhibition of apoptotic protein Bax. Furthermore, Bcl-2 and Bcl-XL are upregulated by *P. gingivalis* gigipain’s adhesin peptide A44, which also inhibits Casp9 activation at early stages. Inhibition of Bax impedes cytochrome c (cytc) release from the mitochondria and blocks the cleavage of casp9 and subsequent activation of effector caspase3, which obstructs the mitochondrial intrinsic apoptotic pathway. *P. gingivalis* inhibits P2X7 receptor (P2X7R)/NADPH oxidase-mediated ROS production and subsequent apoptosis by blocking extracellular ATP (eATP) ligation to P2X7R through its ATP-scavenging enzyme, nucleoside diphosphate kinase (NDK). Additionally, *P. gingivalis* induces antioxidant responses by increasing glutathione (GSH) levels intracellularly possibly by upregulating the uncoupling protein 2 (UCP2). Carcinogenic pathways: (**right**). *P. gingivalis* activates protease-activated receptor 4 (PAR4), which in turn activates the ERK1/2/Ets1 and p38/Hsp27 pathways resulting in pro-MMP9 production. *P. gingivalis*’s activation of PAR2 can mediate pro-MMP9 production via the NF-kB pathway. The cleaved active form of pro-MMP9 is MMP9. Metalloproteinase (MMP) families are involved in ECM and basement membrane degradation and enhance invasion in neoplastic cells. *P. gingivalis* through its virulent factor, a cysteine protease termed gingipain, can cleave pro-MMP9 into its active form MMP9. Gingipains consist of arginine-specific protease A (RgpA) and B (RgpB) and a lysine-specific protease (Kgp), which are responsible for the cleavage of pro-MMP9 into MMP9. Apple polyphenol (AP), hop bract polyphenol (HBP) and high-molecular weight HBP (HMW-HBP) are polyphenols that can inhibit the proteolytic activity of gingipains and can inhibit the PAR2/NF-kB release of pro-MMP9. Thus, *P. gingivalis* promotes invasion through gingipain-mediated activation of MMP9. *F. nucleatum*’s virulent factor FadA binds to the extracellular domain 5 (EC5) of E-cadherin receptor and activates β-catenin that stimulate cyclin D1 and Myc upregulation and binds to T-cell factor/lymphoid enhancer factor (TCF/LEF) to stimulate the secretion of Wnt7a, Wnt7b and Wnt9a, all of which promote cellular growth and proliferation. β-catenin enhances the production of cytokines IL-6, IL-8 and IL-18 and NF-kB1/2 and promotes inflammation, an optimal microenvironment for the prosperity of cancerous cells. *F. nucleatum* and *P. gingivalis* can activate toll-like receptor 2 (TLR2) and mediate TNF-α and IL-6 cytokine production via the TLR2-NF-kB pathway. IL-6 stimulates the activation of the IL-6 receptor (IL-6R), which in turn activates STAT3, known to ultimately upregulate the production of cyclin D1 and promote cellular proliferation.

Based on the information gathered, we propose a model depicting the various pathways involved in *P. gingivalis* and *F. nucleatum*’s pathogenesis in tumor development and progression in neoplastic and non-neoplastic cells (Figure 1).

### 2.2. The Impact of RANKL–RANK–OPG Signaling in PD

Inflammation in PD is inevitably associated with bone lesions leading eventually to teeth loss. At the cellular level, bone lesions are caused by the osteoclast activity, while at the molecular level, the RANK–RANKL–OPG axis is the key player in this process [73]. However, besides bone metabolism, RANKL signaling plays a pivotal role in the immune system development and function. Furthermore, RANKL can exert its effect in a juxtacrine fashion during cell-to-cell contacts (membrane-bound), as well as in a paracrine fashion when it diffuses in body fluids (soluble form). The latter one is a particularly interesting factor in diseases such as PD since it can have systemic effects and could participate in the development of accompanying disorders like osteoporosis or cancer. Therefore, understanding RANKL signaling in bone metabolism and immunity is crucial to infer its role as a potential linker between PD and cancer.

#### 2.2.1. RANK–RANKL–OPG Axis in Bone Metabolism and Immunity

It is a three-component mechanism composed of the receptor RANK (receptor activator of nuclear factor κ B, *TNFRSF11A*), its ligand RANKL (receptor activator of nuclear factor-kappa-Β ligand, *TNFSF11*) and a soluble decoy receptor OPG (osteoprotegerin, *TNFRSF11B*). The RANK–RANKL–OPG axis was discovered in a series of experiments over two decades ago [74,75,76]. In a simplified model of action, RANKL binds to its receptor RANK on the preosteoclasts, inducing osteoclastogenesis and subsequent bone resorption [77]. This effect can be alleviated by high levels of soluble decoy receptor OPG. Experiments in transgenic mice corroborate these models as RANKL- or RANK-deficient (-−/−) mice show osteopetrosis and OPG −/− mice develop the early-onset osteoporosis [78,79,80]. Together, RANK, RANKL and OPG compose the key pathway regulating bone metabolism and turnover in mammals and other vertebrates [81,82,83].

In parallel to its role in bone metabolism, the importance of RANK–RANKL–OPG signaling in the immune system has emerged. Experiments with the use of transgenic mice revealed that RANKL- or RANK-deficient mice show lymph node agenesis and defects in T cell development [84,85]. Further studies confirmed the role of RANKL-RANK in T cell maturation and shaping autoimmunity through the regulation of thymic development [86,87,88]. The binding of RANKL to RANK results in the activation of NF-kB in dendritic cells and enhances their potential to stimulate T cells [89]. In T cells, RANKL binding stimulates JNK kinase, which is required for the effector function of T cells [90,91]. Therefore, RANKL is frequently upregulated in activated T cells and B cells and can mark dendritic cells interacting with T cells [92,93,94]. Therefore, RANKL signaling plays a pivotal role not only in the development of the immune system or maturation of T cells but also in T cell function and inflammatory responses [95].

#### 2.2.2. RANKL Isoforms and Function

The majority of RANKL is present in the form of membrane-bound protein presented on the surface of numerous cells such as T and B lymphocytes, mesenchymal cells, chondrocytes, osteoblasts, osteocytes and megakaryocytes [96]. In adult human tissues, specific staining for RANKL could be detected in bone (osteoblasts), lungs (alveolar cells), sweat glands (epithelial cells), tonsils (crypt epithelium), lymph nodes (lymphocytes and histiocytes) as well as in macrophages scattered within various tissue types [97]. The highest staining intensity was observed in lymph nodes, which is not surprising, considering the high abundance of B cells, dendritic cells and proliferating T cells, all expressing RANKL [97,98]. Immunohistochemical methods detect membrane-bound RANKL, however, there is also a fraction of RANKL referred to as soluble RANKL (sRANKL). The soluble form is produced, inter alia, by the bone marrow stromal cells (ST2 cell line) and stimulated memory B cells [93,99]. Moreover, pathological cells such as multiple myeloma cancer cells can overexpress sRANKL, which results in their impact on the skeletal system of patients [100]. Another pathway for the production of the sRANKL is its cleavage by membrane-bound proteinases such as TACE, ADAM10 and MMP14, with the latter having the highest contribution to the release of sRANKL [82,101]. It is worth noting that both forms of RANKL, i.e., membrane-bound and soluble, are inducing osteoclast differentiation, although the effect of membrane-bound form is stronger [76]. The major difference between the two isoforms is their range of action. The membrane-bound form acts through direct, cell–cell contacts whereas the soluble form acts through paracrine diffusion and can have systemic effects. For instance, sRANKL can serve as an attractant for cells as it was shown for monocytes, osteoclasts and regulatory T cells [102,103,104].

#### 2.2.3. Role of RANKL in Periodontal Disease

Bone erosion in PD leads to teeth loss and for a long time this was the major, dishonorable role of RANKL in the development and progression of the disease. However, recent studies revealed that RANKL signaling has a more complex role in the disease and that tooth loss is an evolutionary mechanism eliminating a source of inflammation [105]. The disease begins with the pathogen-induced destruction of the barrier formed by gingival epithelial cells followed by infiltration of leukocytes such as macrophages, dendritic cells and T cells [106,107]. Experiments using the mice model of PD revealed that indeed, the expression of proinflammatory cytokines such as IL1B, IL17 and TNF is high at the early stage of the disease [104]. After 28 days of disease progression, the proinflammatory gene expression profile is replaced by a wound-healing gene expression marked by high levels of *OPG*, *COL5A1*, *CTGF*, *FGF7*, *ITGA4*, *ITGA5* and *SERP1*. This also correlates with increased numbers of FOXP3+ regulatory T cells in the surrounding tissue, which downregulate inflammation, allow wound healing and suppress osteoclastogenesis [104,108]. Treatment with an antibody raised against RANKL alleviates wound healing and FOXP3+ cell recruitment, indicating that sRANKL acts as a cytokine attracting Treg cells to the inflammation site. However, when the immune system is incapable of defeating the infection, the presence of Th17 cells expressing membrane-bound RANKL stimulates osteoclastogenesis, bone erosion and eventually tooth loss, removing the source of inflammation [105,109].

One of the consequences of PD that could have systemic effects is an increased level of sRANKL in the blood serum [110]. In addition to the blood serum, high levels of sRANKL were observed in tissue homogenates, gingival cervical fluid (GCF) and saliva [92,111,112]. It was suggested that sRANKL is produced mostly by T and B cells infiltrating the diseased gingival tissue [92]. However, sRANKL can also be produced by the action of matrix proteinases such as MMP14 and TACE [101,113]. Interestingly, despite the in vitro results suggesting a low activity of TACE towards RANKL [114], recent studies underscore the role of this protease in the release of sRANKL and osteoclastogenesis in PD [113]. Furthermore, the expression of TACE is increased in PD and the enzyme is found in the GCF of PD patients, which corroborates its involvement in high sRANKL levels in serum and other body fluids [115,116].

Reports showing levels of sRANKL in serum in human disease are surprisingly scarce and mostly focused on bone-related diseases such as PD or osteoporosis. Both diseases are characterized by increased serum sRANKL, as well as increased risk of occurrence of PD in osteoporosis and vice versa, that is, the development of osteoporosis in PD [29,117]. In addition to bone-related disease, elevated levels of sRANKL in serum are also observed in other diseases such as rheumatoid arthritis [118], coronary artery calcification [119], Paget’s disease of bone [120] and cancers such as multiple myeloma [120,121], neuroblastoma [122], prostate cancer [123] or breast cancer [124]. In cancer, it is unclear if the high level of sRANKL is a cause or an effect of the disease. Breast cancer, prostate cancer and multiple myeloma are characterized by high expression of *RANKL* [125,126,127]. Noteworthy, high expression of *RANKL* is associated with a high incidence of metastases and poor survival prognosis in patients with the aforementioned types of cancer [128,129]. To date, it was shown that high levels sRANKL could perceive the development of type II diabetes [130] and the risk of developing breast cancer [131]. However, more detailed studies are needed to determine whether high sRANKL levels can be the cause of human diseases other than osteoporosis.

#### 2.2.4. RANKL Links Periodontal Disease and Cancer

**Figure 2:**
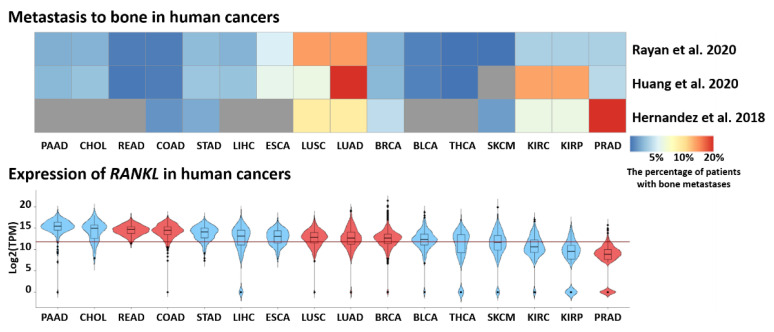
Bone metastasis and *RANKL* expression in human cancers. The upper panel shows the percentage of patients with bone metastasis at the cancer diagnosis time. Studies utilized Surveillance, Epidemiology and End Results (SEER) and Oncology Services Comprehensive Electronic Records (OSCER) databases [139,140,141]. The percentage of patients is presented as a heatmap with the color intensity corresponding to higher percentage of cases. Missing data for selected cancer types are in gray. The lower panel shows the expression of *RANKL* (*TNFSF11*) in selected primary tumors. Violin plots show log2-transformed expression of *RANKL* gene in selected cancer types. Cancers with the confirmed role of RANKL–RANK signaling in metastases are marked in red. The horizontal red line shows the median expression for all cancers in the dataset. Abbreviations: PAAD—pancreatic adenocarcinoma, CHOL—cholangiocarcinoma, READ—rectum adenocarcinoma, COAD—colorectal adenocarcinoma, STAD—stomach adenocarcinoma, LIHC—liver hepatocellular carcinoma, ESCA—esophageal carcinoma, LUSC—lung squamous cell carcinoma, LUAD—lung adenocarcinoma, BRCA—breast invasive carcinoma, BLCA—bladder carcinoma, THCA—thyroid carcinoma, SKCM—skin cutaneous melanoma, KIRC—kidney renal clear cell carcinoma, KIRP—kidney renal papillary cell carcinoma, PRAD—prostate adenocarcinoma. Data for GDC Pan-Cancer (PANCAN) dataset were downloaded from the UCSC Xena portal (https://xena.ucsc.edu/, accessed on 20 November 2021).

The most recent studies confirm a higher risk of breast cancer occurrence in PD and show an increased risk for other cancers such as head and neck cancer, colorectal cancer and lung cancer [2,25,132,133,134]. Interestingly, breast, colorectal and lung cancers have been shown to have high metastatic potential associated with RANKL–RANK signaling [135,136]. Furthermore, there is constantly accumulating experimental and epidemiological evidence underscoring the role of the RANKL–RANK pathway in cancer survival and metastasis [135,137,138]. Not surprisingly, *RANKL* expression is elevated in a number of primary solid tumors (Figure 2), which frequently metastasize to bones such as lung squamous cell carcinoma, lung adenocarcinoma or breast cancer. It was also observed that metastatic prostate tumors show higher expression of *RANKL* than nonmetastatic primary tumors, further underscoring a role of RANKL in tumor dissemination [126]. Therefore, RANKL signaling could provide a molecular link between periodontal disease and the prevalence of cancer or cancer metastasis.

One of the possible mechanisms acting in favor of cancer development in PD is the generation of the immunosuppressive microenvironment. Serum levels of sRANKL are elevated in PD patients and recent studies show that sRANKL is the key cytokine in immune response modulation, acting through the recruitment of Treg cells and the transition between Th17 and FOXP3+ regulatory cells [104,108]. The attraction of Treg cells creates an immune-suppressive microenvironment that can allow the survival of malignant cells and is frequently observed in solid tumors [142]. In tumors, excessive stimulation of tumor-infiltrating T cells results in exhaustion phenotype, reduced proliferation, responsiveness or cytotoxicity of T cells [143]. Interestingly, the upregulation of exhaustion markers such as LAG-3 and TIM-3 is also observed in PD, suggesting that the chronic inflammation in gingival tissue shares immune phenotypes with tumor microenvironment [144]. In addition to Treg accumulation, periodontal inflammation recruits myeloid-derived suppressor cells [145], suggesting that chronic inflammation in PD creates a multicellular, immunosuppressive microenvironment, similar to the microenvironment of tumors. Further studies are required to elucidate whether gingival immunosuppressive foci in PD could be associated with tumorigenesis.

Another possible mechanism linking PD, sRANKL and cancer is metastasis. Recent studies show that sRANKL plays a crucial role in bone metastatic cancers [135,146,147]. Experiments with sRANKL-deficient mice (Tnfsf11ΔS/ΔS) revealed significantly decreased bone metastasis in melanoma and breast cancer models [148]. Another recent study also showed the involvement of RANKL in metastases of colorectal cancer (CRC) cells expressing high levels of RANK [136]. Interestingly, immunofluorescent staining revealed that CD3+CD25+FOXP3+ Treg cells were colocalized together with RANKL+ cells in CRC tissue. A similar phenomenon was observed in breast cancer metastasis suggesting that CD4+CD25+FOXP3+ Treg cells are the major source of RANKL-promoting metastasis [149]. It is unclear exactly how RANKL produced by Treg cells could lead to bone lesions and metastases, especially considering the fact that Tregs can suppress osteoclastogenesis in PD [108]. However, it is likely that membrane-bound RANKL present on tumor-infiltrating lymphocytes is being cleaved by proteases and released to the bloodstream where it acts in a paracrine manner. By promoting bone resorption and the formation of bone lesions, sRANKL in PD could create potential entry sites for metastatic cancers. While the exact mechanism linking sRANKL with cancer needs to be elucidated, the importance of sRANKL for cancer dissemination is underscored by clinical trials with denosumab adjuvant therapy showing a reduced incidence of skeletal metastases in multiple tumors [150,151]. However, controlling sRANKL levels with the antibody in PD is associated with frequent denosumab-related osteonecrosis of the jaw (DRONJ) and further studies are required to optimize the treatment or develop alternative control strategies [152].

### 2.3. Periodontal Disease and Immune Response

#### 2.3.1. Periodontal Disease and Alterations in Blood: The Impact on Systemic Diseases

Periodontal disease should not be only perceived as localized inflammation. It has been proven that any sort of irritation to the inflamed gingiva, even such as daily brushing and flossing, can cause transient bacteremia and in some cases a distant infection [153]; e.g., monocytes/macrophages activated by periodontitis adhered to vascular endothelium, starting the process of arteriosclerosis and leading to aortic inflammation [154]. Inflammatory mediators, cytokines, chemokines, bacterial toxins, fragments of bacteria and bacteria themselves can enter the bloodstream directly, via lymph, transported by lymphocytes [155].

The model of experimentally induced inflammation of the gingiva has shown that it has systemic consequences. Increased levels of CRP, IL-6 and MCP-1 were observed in the blood [156]. The same observations have been reported in patients diagnosed with PD. Proinflammatory cytokines such as IL-1β, IL-2, IL-8 and CRP were also in abundance in the systemic circulation [157]. Conversely, other studies have generated contradictory data. Cheng et al. focused on the influence of periodontal disease on immune cells subsets and cytokines present in the peripheral blood by comparing the blood samples collected from patients with chronic or aggressive periodontitis with samples from participants with healthy periodontium [145]. There were no significant differences between CD4+, CD8+ and γδ T cells, CD19+ B cells, CD14+ monocytes and CD56+ NK cells subsets among the three groups; CD45RA+ or CD45RO+ cells within CD4+ T cell population were similar amongst them. CD markers (CD14, CD16, CD40, CD54, CD86 or HLA-DR) expressed on CD14+ monocytes were not significantly different between the groups either. Although CD4+ T cells expressed more TNF-α in comparison to anti-inflammatory IL-10 in chronic periodontitis, there was no significant difference when collated with the other groups. The results for CD14 and CD16 are inconsistent with another study, which showed that an increased percentage of CD14+CD16+ monocytes was observed in the blood in chronic periodontitis [158]. Analogically, even though levels of IL-4, IL-6, IL-10, IL-17F, IL-33 and TNF-α were elevated in the serum obtained from patients with chronic periodontitis, there were no significant differences in comparison to healthy subjects. The level of IL-17 in the peripheral blood has been noticed to increase during ongoing periodontal disease and drop after treatment [159]. There are also studies suggesting that a consensus on the matter of changes in serum levels of INF-γ, IL-4 and IL-17 has not been reached [145]. This variability amongst studies shows that new data and meta-analyses summarizing and validating changes in blood and tumor microenvironment adjusted for PD should be considered. A focus on every single molecule might be necessary.

In their cross-sectional study on a potential correlation between coronary artery disease and periodontal disease Kampits et al. analyzed IL-1β, IL-6, IL-8, IL-10, IFN-γ and TNF-α levels in the blood samples obtained from patients with stable coronary artery disease who met the study criteria. After classifying the severity of the periodontitis among participants it was observed that the levels of cytokines such as IFN-γ, IL-10 and TNF-α were elevated in PD. However, there was no association between IL-1β, IL-6 and IL-8 concentrations and PD [160].

**Figure 3:**
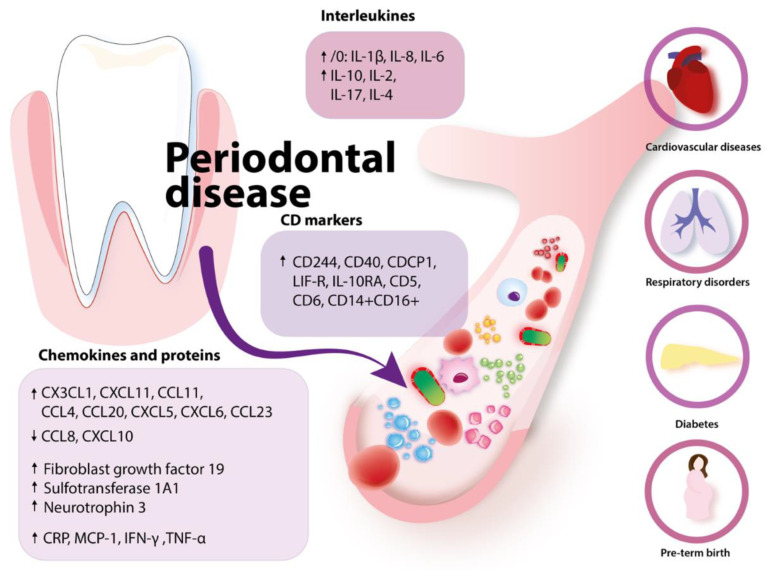
The inflammatory response in periodontal disease and its systemic consequences. The figure illustrates changes in chemokines, proteins, interleukins levels and CD markers present on lymphocytes in the periodontal disease. Inflammatory molecules enter the bloodstream and increases the susceptibility of the organism to systemic diseases like cardiovascular, respiratory, metabolic diseases and pregnancy problems.

Pregnancy complications such as preterm delivery may also be the result of circulating cytokines connected to active periodontitis. A pregnant woman with diagnosed periodontal disease had increased levels of IL-2, IL-4, IL-6, IL-10, TNF-α and INF-γ [159]. Moreover, in the study of Panezai et al., the authors analyzed three cohorts of patients, one with PD (n = 38), the second with rheumatoid arthritis (n = 38) and the third with healthy subjects (n = 14) [161]. The study reported the positive correlation of chemokines CCL8, CX3CL1, CXCL10, CXCL11, CCL11, CCL4, CCL20, CXCL5, CXCL6, and CCL23 with the number of teeth. Chemokines such as CCL8 and CXCL10 were inversely associated with marginal bone loss (MBL). CD markers such as CD244, CD40, CDCP1, LIF-R, IL-10RA, CD5 and CD6 were significantly related to bleeding on probing (BOP), MBL, number of teeth and shallow and deep pockets. In addition, some inflammatory proteins like fibroblast growth factor 19, sulfotransferase 1A1 and neurotrophin 3 were positively correlated with BOP, probing pocket depth and MBL [162]. In general, the mentioned molecules are involved in different proinflammatory processes and may be treated as the indicators of systemic response [162,163,164]. It was also shown that CD5 and CD6 levels (surface molecules expressed on both T and B lymphocytes) are significantly associated with the increased number of deep pockets, which is an important parameter determining the severity of PD [162,165]. The level of CD6 was also correlated with MBL [162]. We summarized the congregated data in Figure 3.

It is worth pointing that some studies’ statistical analyses were based on comparing differences amongst some cytokines or circulating cells levels, as an approach to demonstrate significant pathological implications between healthy controls and patients with PD. A more specific and direct approach was used by investigating a correlation between PD parameters such as clinical attachment loss and probing death and alterations in the blood. The application of different approaches and the lack of consistency may result in diversified conclusions and difficulties in the comparison of studies. In studies evaluating some changes in blood during PD, both direct comparison (healthy subjects vs. PD patients) and parameters-based correlations should be made in order to provide a complete picture of variability.

#### 2.3.2. Potential Mechanisms Linking with Cancer

Although some alterations of cytokines and immune cells in PD were observed, up to date little is known about their impact on carcinogenesis. There are many unanswered questions due to the lack of models investigating the interaction between PD and cancer. Nevertheless, the literature indicates that cancer and its treatment affect the systemic immune response, which in turn affects cancer development and can be considered an important player in prognosis [166,167,168,169,170,171,172].

As it was mentioned above, a significant change in cytokine levels or immune cells number were observed in PD. For example, two of them were increased CD5 and CD6 levels [162]. CD5 and CD6 molecules play a role in cancer and chronic lymphocytic leukemia [173,174,175]. The CD5 has an inhibitory function in T and B1a cell activation [173]. However, its different alleles influence the outcome and mortality in melanoma in opposite direction, depending on its capacity to downregulate TCR-mediated intracellular signals [174]. For CD6, there is still a lack of studies investigating associations with malignancies. Interestingly, the study based on mouse models reported that the absence or blockade of CD5- and CD6-mediated signals resulted in the dysfunction of the immune response, subsequently enhancing cancer progression [175]. Based on the small number of data associating CD5 and CD6 with cancer, we cannot draw a clear conclusion and thus a question remains in mind: are they a potential link between PD and cancer? Certainly, the role of CD5 and CD6 in PD-associated cancers should be furtherly investigated. Another example may be Il-17, whose level was increased in ongoing PD [160]. Increased levels of Il-17 promoted the development and epithelial-to-mesenchymal transition in prostate cancer and others [175]. A similar question to the one pertaining to CD5 and CD6 can be raised regarding IL-17.

Only one of the available studies analyzed directly common PD and cancer model [145]. It was shown that PD significantly correlated with a greater frequency of lymph node micrometastases. Surprisingly, the authors observed that injection of LPS in the peritoneal cavity did not stimulate metastatic response, whereas a sterile model of periodontal inflammation induced metastasis. Moreover, PD seemed to attract metastases directly toward the inflammation site [145]. The authors focused on macrophages and myeloid-derived suppressor cells (MDSC), which were significantly more numerous in lymph nodes in comparison between mice with and without PD. It was reported that MDSC may be recruited in the periodontal inflammation, being induced by *Porphyromonas gingivalis* infection [145,176]. Both M2 and M1 were recruited to the inflammation site. Macrophages migrated to tumor niche, leaving the spleen, and were more numerous during infiltration in mice with PD. Moreover, it was proven that IL-1β is a major player that promotes CCL5, CCL2, CXCL5 and CXCL12 expression. Mainly, these chemokines participate in MDSC and macrophages recruitment process, creating the proinflammatory microenvironment that is a niche for metastasis of breast cancer cells the in place of inflammation [145]. The study of Cheng is one of the first trying to understand the mechanism. In the literature, there are numerous meta-analyses proving the association between PD and cancer risk, but there is a lack of mechanism-focused analytic studies.

## 3. ClinicalTrials.gov Analysis

In order to provide an insight into clinical trials linking PD and cancer, the registry ClinicalTrials.gov (accessed on 3 November 2021) was analyzed. For the search, the term “periodontal disease and cancer” was used. Then, 5 of the 20 searched trials were selected for analysis. The number of studies investigating PD in the context of cancer is still highly limited. In addition, all of the analyzed studies focus only on oral cancer (n = 3) and breast cancer (n = 2). In contrast, the term “periodontal disease and cardiovascular disease” produced 42 results. The studies investigate the increased predisposition to oral cancer incidence in the presence of PD, the etiology and mechanism of postradiotherapy dental disease, the viral etiology of PD in relation to radiotherapy of head and neck cancers and the impact of aromatase inhibitors on oral health and quality of life. Only one study (NCT03244943) that evaluates cytokine profiles in breast cancer patients during systemic chemotherapy treatment may give a more precise insight into potential mechanisms linking the PD severity and cancer prognosis. The authors adjusted this analysis to some dental parameters such as, e.g., probing depth, assessing the impact of nonsurgical periodontal therapy and related remission of PD on cytokine profile and final outcome of patients. As we suggested in our study, new studies assessing the mechanism may account for discovering new treatment methods or prognostic and diagnostic biomarkers. There is a need for clinically orientated trials specifically focusing on PD and cancer associations. A high number of analyzed subjects is necessary in order to detect some alterations. We summarized and detailed all analyzed studies in Table 2.

## 4. Conclusions and Future Perspectives

Periodontal disease is an inflammatory disease with systemic effects leading to the development of secondary health complications, among them cancer. In cancer, it is still unclear whether PD is a cause or a consequence of cancer, since there are studies supporting both hypotheses. This review provides an overview of recent discoveries and putative mechanisms linking PD and cancer development and progression. Herein, the three prominent hallmarks of PD are presented: the direct effects of bacterial infection, the RANKL signaling pathway and the systemic effects of cytokine signaling. We highlighted the role of the etiological factor of PD, namely the infection with *P. gingivalis* and *F. nucleatum* that is directly involved in tumorigenesis of OSCC through suppression of apoptotic pathways, stimulation of prosurvival signals in gingival epithelial cells and interfering with the cell cycle. An interesting common ground between PD and cancer could also be the RANKL–RANK signaling pathway, which is frequently disrupted in both diseases. RANKL cytokine is required for attracting regulatory T cells to the inflammation site. While this mechanism physiologically leads to the extinguishing of the inflammation, it can have side effects in creating an immunosuppressive microenvironment. Moreover, RANKL signaling is also associated with cancer metastasis and since sRANKL is elevated in PD, it may be the factor responsible for tumor cell dissemination and metastasis. Another systemic consequence of PD is an alteration of proinflammatory cytokines levels, which could influence the immune response and tumor microenvironment. In particular, cytokines could promote the migration and dissemination of MDS cells, which can enable tumor growth. While the above mechanisms have proven general roles in cancer development, their exact influence in the course of PD needs to be further investigated. Specifically, more studies are required to elucidate whether bacterial factors could be associated with nonoral cancers or to assess whether PD-associated RANKL secretion leads to tumorigenesis and tumor dissemination. In addition, more longitudinal studies are needed to determine whether PD is a cause or a consequence of cancer.

## Figures and Tables

**Table 1:** Mechanisms of systemic diseases associated with periodontal diseases.

Health Problems Associated with Peridontal Disease	Mechanism
Cardiovascular	Elevated acute phase proteins (CRP, haptoglobin, alfa1-antitrypsin, fibrinogen) due to periodontitis Elevated CRP is associated with a higher risk of myocardial infarction and peripheral artery disease
Diabetes	Progression of diabetes: IL-1β and TNF-α increase insulin Endotoxins or LPS → inflammation
Respiratory	Aspiration of oral bacteria Enzymes secreted with saliva in periodontal disease may change mucosa and lead to higher adhesion and colonization of respiratory microbes Enzymes secreted in periodontal disease by *P. gingivalis* degrade salivary elements that bind pathogens and preclude them from mucosal adhesion Cytokines secreted in periodontal disease may modify respiratory epithelium
Problems with pregnancy	Low birth weight Preterm birth: elevated LPS stimulates placenta calls to secrete IL-1β and PGE-2
Rheumatoid arthritis	*P. gingivalis* synthetizes citrullinated proteins causing the organism to produce anti-citrullinated proteins antibodies

**Table 2:** Studies which are registered in ClinicalTrials.gov system regarding periodontal diseases and cancer aspects. ND—no data; NA—not applicable.

Title of Project	ClinicalTrials.gov Identifier/Current Status	Condition	Number of Particiapnts (n)	Intervention	Primary Outcome	Secondary Outcome	Country
The Link Between Periodontitis, Smoking and Oral Cancer	NCT04047212/not yet recruiting	Chronic periodontitis Oral cancer	200	Diagnostic test: biopsy for oral cancer Diagnostic test: periodontal examination	Periodontitis: occurrence of periodontitis or increase in the grade of an already existing case of periodontitis	Oral cancer: occurrence of a lesion of oral cancer or a premalignant lesion	Egypt
Postradiation Dental Disease Amongst Head and Neck Cancer Patients	NCT03703648/recruiting	Head and neck cancer Caries Periodontal diseases Radiotherapy side effect	215	Radiation: radiotherapy (curative) for head and neck cancer	Dental caries: the mean number of carious teeth amongst head and neck cancer patients postradiotherapy	Periodontal disease: the proportion of head and neck cancer patients with periodontal disease postradiotherapy. Xerostomia measured using the Xerostomia Questionnaire (XQ): change from baseline. Range of scores from 0 (no xerostomia) to 90 (worst xerostomia) Oral health Quality of life: change from baseline. Fourteen oral health problems presented—patients asked to score how often they are personally encountered (very often, fairly often, occasionally, hardly ever, never, do not know) Salivary flow rate: change from baseline Mouth opening: change from baseline Diet assessed by question 13 of the World Health Organisation’s Oral Health Questionnaire for Adults: change from baseline. Respondents asked to detail how often they consume, e.g., sugar buns Oral hygiene practice assessed by questions 7, 8, 9 of the World Health Organisation’s Oral Health Questionnaire for Adults: change from baseline. Patients asked to indicate, e.g., how often they brush their teeth Tooth loss Costs of treatment to patients and NHS	UK
Cytokine Profiles in Breast Cancer Patients Undergoing Chemotherapy	NCT03244943/completed	Mammary neoplasm, human Periodontal diseases Chemotherapy effect	40	Procedure: nonsurgical periodontal treatment	Cytokines: cytokine levels and changes before and after posttreatment	Correlation of cytokines: cytokine levels between parameters clinical	Brazil
Oral Health in Breast Cancer Survivors on Aromatase Inhibitors	NCT01693731/completed	Periodontal disease Quality of life	300	ND	Periodontal diseases	Alveolar bone loss using salivary and serum-derived bone markers. Oral Health Related Quality of Life (OHRQoL) assessed via questionnaire	USA
Towards a Viral Etiology of Periodontal Disease in Relation to Radiotherapy Treatment of Head and Neck Cancers	NCT02180932/completed	Periodontal disease	25	Biological: periodontal pocket samples	Measure of level of EBV nucleic acids Measure of level of EBV nucleic acids	NA	France
